# The causal effect of inflammatory bowel disease on diffuse large B-cell lymphoma: two-sample Mendelian randomization study

**DOI:** 10.3389/fimmu.2023.1171446

**Published:** 2023-08-01

**Authors:** Chuanyang Lu, Qiuni Chen, Hong Tao, Lei Xu, Jiaxin Li, Chunling Wang, Liang Yu

**Affiliations:** ^1^ Department of Hematology, The Affiliated Huaian No. 1 People’s Hospital of Nanjing Medical University, Huaian, China; ^2^ Key Laboratory of Hematology of Nanjing Medical University, Nanjing, China

**Keywords:** inflammatory bowel disease (IBD), diffuse large B-cell lymphoma (DLBCL), genome-wide association studies (GWAS), single-nucleotide polymorphisms (SNPs), Mendelian randomization (MR)

## Abstract

**Background:**

It has been reported that inflammatory bowel disease (IBD) is associated with an increased risk of malignancies, including lymphoma. A number of large observational studies have been devoted to exploring the causal link between IBD and malignant lymphoma. However, no consensus exists on whether there is a causal relationship between IBD and malignant lymphoma.

**Methods:**

The summary dataset of the IBD and lymphoma genome-wide association studies (GWAS) was obtained from the OPEN GWAS website. Single-nucleotide polymorphisms (SNPs) were selected as genetic instrumental variants (IVs) for fulling P < 5 × 10^-8^ and linkage disequilibrium (LD) of r^2^ = 0.001 in the IBD GWAS. The proxy SNPs with LD of r^2^ > 0.8 were identified. Palindromic SNPs and outlier SNPs were excluded. The assessments of sensitivity employed the Cochran’s Q test, Mendelian randomization (MR)-Egger intercept test, and leave-one-out analysis.

**Results:**

The MR analysis results proved the causality of IBD on diffuse large B-cell lymphoma (DLBCL). The risk of developing DLBCL is increased by 28.6% in patients with IBD [odds ratio (OR)_IVW_ = 1.286, 95% confidence interval (CI) 1.066–1.552, P = 0.009]. The results of the subgroup analysis showed that Crohn’s disease (OR_IVW_ = 1.218, 95% CI 1.030–1.441, P = 0.021) rather than ulcerative colitis (OR_IVW_ = 1.206, 95% CI 0.984–1.478, P = 0.072) had a causal effect on DLBCL. No horizontal and directional pleiotropy was observed in the MR studies.

**Conclusions:**

The above MR study concluded that IBD itself is causally responsible for DLBCL, especially Crohn’s disease. Further investigations are needed to elucidate the mechanism underlying this direct causal link.

## 1 Introduction

Malignant lymphoma is a neoplasm of the lymphatic system that is categorized according to the origin of the cells and stage of differentiation. The two main types of lymphoma are Hodgkin’s lymphoma (HL), which is derived from B cells, and non-Hodgkin’s lymphoma (NHL), which is derived from B cells and T cells (1). There are many factors associated with lymphoma development. The most well-known of them is chronic inflammation (2). Inflammatory bowel disease (IBD) is a bunch of immune-mediated advanced inflammatory conditions of the gastrointestinal tract characterized by a chronic inflammation of the intestine (3). The two major entities of IBD are Crohn’s disease and ulcerative colitis (4).

There is suggestive evidence that IBD patients have an increased risk of lymphoid tumors. Two main factors contribute to that: the inflammatory process itself and the widespread use of immunomodulators (5). Research has demonstrated that being elderly (>65 years old), the male gender, and the use of thiopurine are critical factors that increase the risk of developing lymphoma (6, 7). Meanwhile, for patients with the absence of anti-tumor necrosis factor (TNF) and/or thiopurine, the risk of developing lymphoma is relatively decreased (8). Whether a causal association between IBD and lymphoma exists is controversial (8, 9).

In this study, we aimed to investigate the casual effect of IBD on the risk of developing malignant lymphoma by Mendelian randomization (MR) analysis of the summary statistics of genome-wide association study (GWAS) datasets.

## 2 Materials and methods

### 2.1 Data sources for IBD and lymphoma GWAS

The summary dataset of the IBD GWAS was obtained from the International IBD Genetics Consortium (IIBDGC) consortium, as determined by Liu et al. (10). A sample of 65,642 individuals of European ancestry was enrolled in the study, including 31,665 cases and 33,977 controls. Of the 31,665 patients with IBD, there were 13,768 patients with ulcerative colitis and the remaining were Crohn’s disease patients. The GWAS databases for malignant lymphoma were available from the FinnGen website (https://www.finngen.fi/en). The GWAS data associated with HL consisted of 369 cases and 180,756 controls. The GWAS data correlated with NHL included diffuse large B-cell lymphoma (DLBCL; 209 cases), follicular lymphoma (FL; 522 cases), mature T/NK-cell lymphomas (150 cases), and other and unspecified types of NHL (533 cases). All of the above GWAS datasets are publicly available and can be downloaded from the OPEN GWAS website (https://gwas.mrcieu.ac.uk/).

### 2.2 Genetic instrumental variant selection for IBD

**Figure 1:**
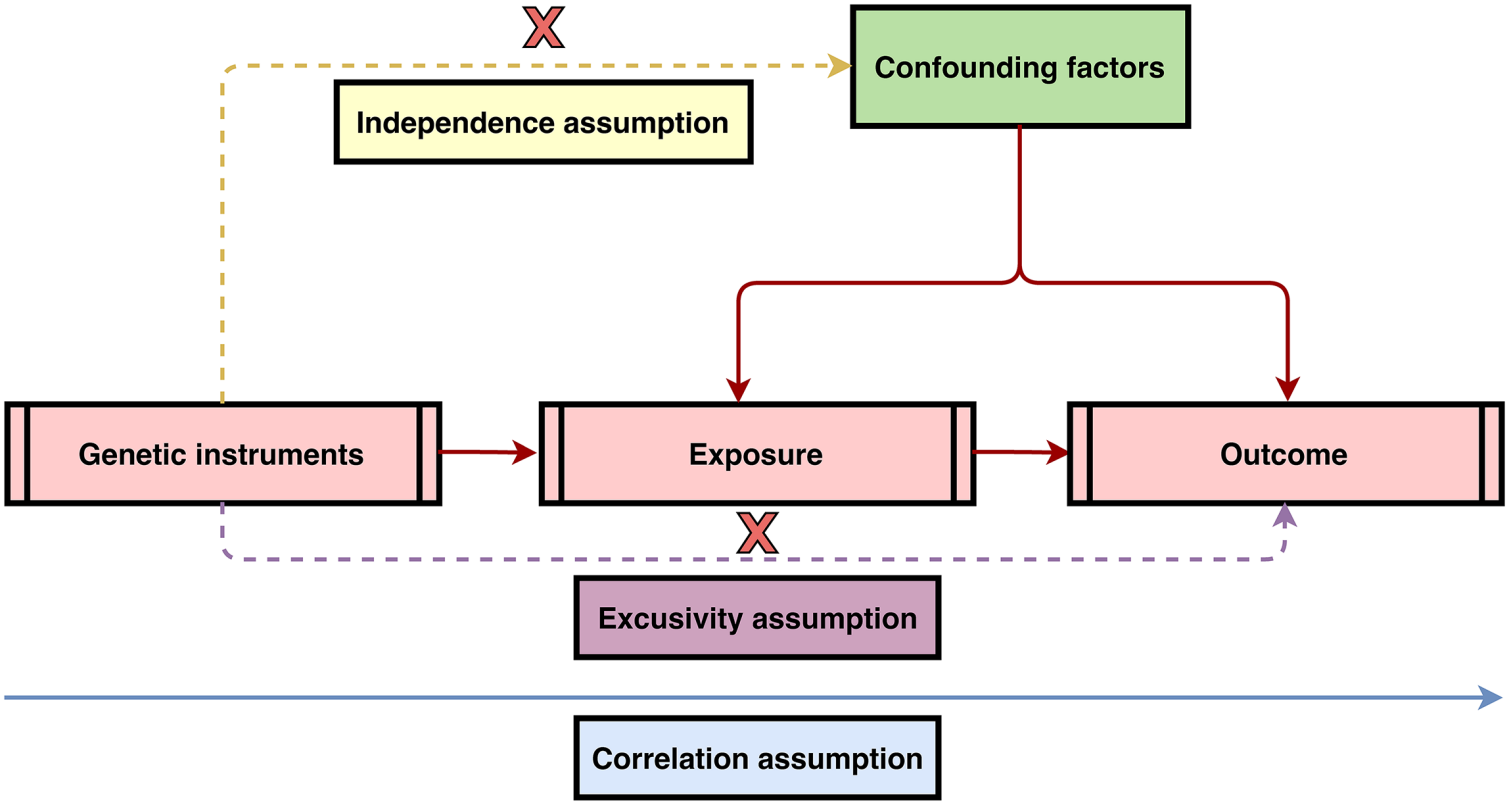
Three core assumptions of Mendelian randomization (MR) analysis. 1) Correlation assumption: there is a strong association between instrumental variants (IVs) and exposure factors; 2) Independence assumption: the IVs are independent of confounding factors of the exposure–outcome relationship; and 3) Exclusivity assumption: genetic variants can only affect the outcome through the exposure and not through other pathways.

MR study designs have to satisfy three core assumptions: 1) there is a strong association between instrumental variants (IVs) and exposure factors; 2) the IVs are independent of confounding factors of the exposure–outcome relationship; and 3) genetic variants can only affect the outcome through the exposure and not through other pathways (11). The above three assumptions were visualized in **Figure 1**. To fulfill the mentioned core assumptions, first, the IVs with genome-wide significance (P < 5 × 10^-8^) were extracted from the IBD GWAS. The linkage disequilibrium (LD) of r^2^ = 0.001 and clumping distance = 10,000. Then, proxy single-nucleotide polymorphisms (SNPs) with LD of r^2^ > 0.8 were identified as well as palindromic SNPs were excluded when harmonizing the IBD and malignant lymphoma GWAS. Next, MR Pleiotropy RESidual Sum and Outlier (MR-PRESSO) test was employed to discern potential outlier SNPs for correcting potential horizontal pleiotropy (12, 13). Finally, the remaining SNPs were utilized for MR analysis. And the F-statistic was calculated for each leftover SNP to measure the strength of genetic IVs. IVs with F-statistic >10 indicate not a weak genetic instrument. In the ulcerative colitis and Crohn’s disease GWAS, genetic variants were screened in the same process.

### 2.3 MR analysis

**Figure 2:**
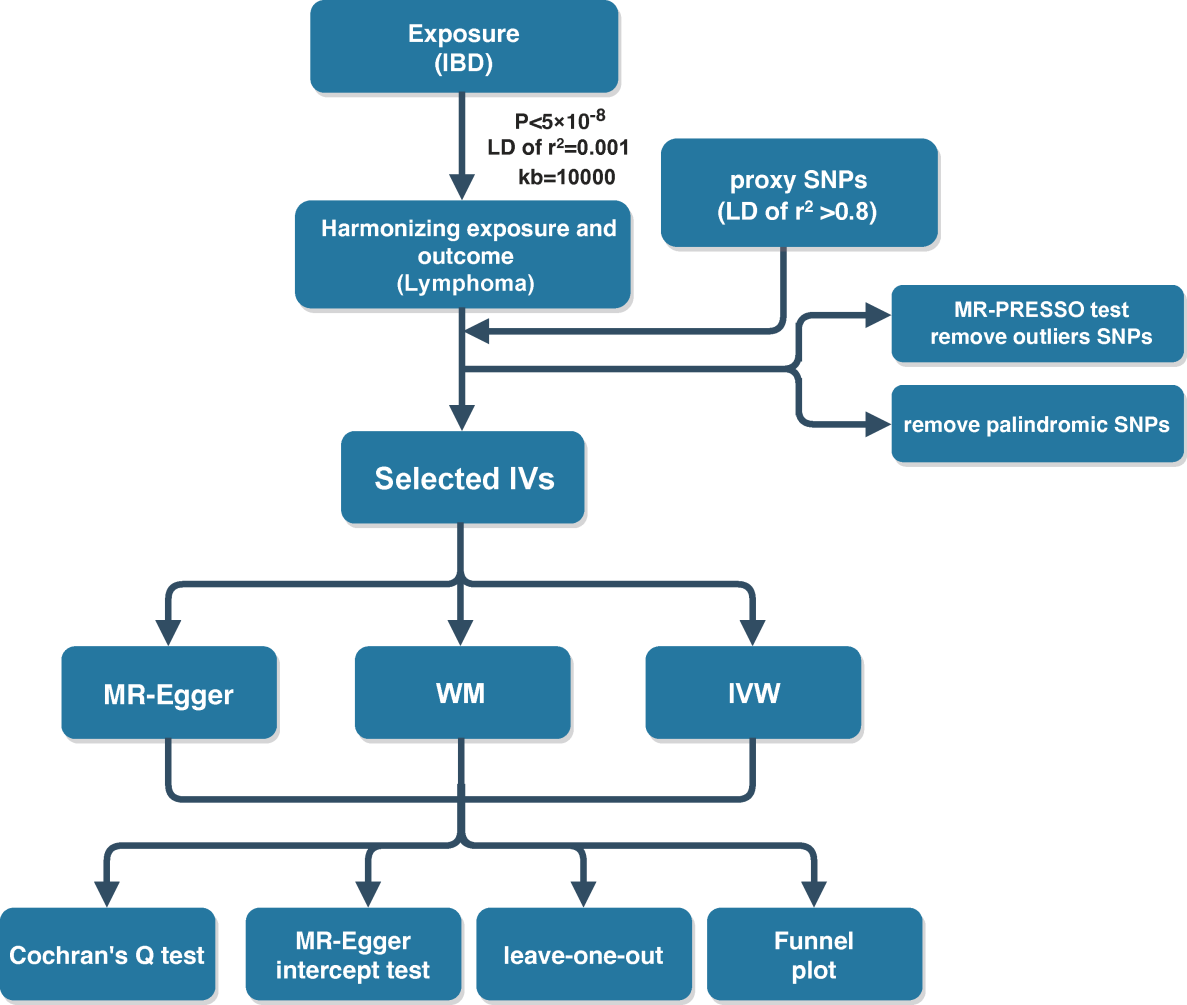
The flow diagram of the Mendelian randomization (MR) study.

Three MR methods including MR-Egger, inverse variance-weighted (IVW), and weighted median (WM) were applied for the estimation of the causality of IBD and lymphoma. The IVW model assumes that all enrolled SNPs are valid genetic IVs and no pleiotropic effects exist (14). If the causal effect estimates of the three models are inconsistent, the results of IVW methods are considered as the main outcome. The odds ratio (OR) was treated as the effect size for the determination of the direction of causality, with OR >1 indicating that the exposure was a risk factor for the outcome. P-values < 0.05 indicated a statistically significant finding of causality. Cochran’s Q test was used to examine the heterogeneity among genetic IVs. The sensitivity analysis was mainly performed by the leave-one-out method. The results of the causal relationship between IBD and malignant lymphoma were observed to remain stable and reliable after deleting one SNP at a time. The MR-Egger intercept test was conducted to detect directional pleiotropy, with P-value <0.05 demonstrating the presence of directional pleiotropy. Funnel plots were plotted to assess directional pleiotropy, similar as its role in meta-analysis to assess publication bias. The MR analysis is implemented in the R software (version 4.1.3) using the R packages “TwoSampleMR” (version 0.5.6) and “MRPRESSO” (version 1.0). The flow schematic of this study was shown in **Figure 2**.

## 3 Results

### 3.1 Genetic IVs for IBD

There were 134 genome-wide significant (P < 5 × 10^-8^) and independent SNPs extracted from the IBD GWAS. When harmonizing the exposure and outcome GWAS, five proxy SNPs with LD of r^2^ > 0.8 were identified because they were not available in the outcome GWAS. A total of eight palindromic SNPs (rs10878302, rs10956252, rs11641016, rs2488397, rs35730213, rs36048684, rs6466198, and rs78487399) and two incompatible SNPs (rs11768997 and rs140143) were removed. After MR-PRESSO test, no outlier SNPs were found from the above selected genetic IVs. Finally, 124 genetic variants that strongly associated with IBD were used for the follow-up MR analysis. The F-statistics of the included instrumental SNPs were all >10, suggesting the absence of weak IVs. The general information of the chosen genetic IVs was described in **Supplementary Table S1**.

IBD includes the two main forms of ulcerative colitis and Crohn’s disease. Studies have shown that ulcerative colitis and Crohn’s disease exhibit strong heterogeneity in terms of incidence patterns, disease localization, disease progression, endoscopic features, and response to treatment (15). Therefore, IBD GWAS was divided into two groups, ulcerative colitis and Crohn’s disease, for subgroup analysis. For ulcerative colitis GWAS, 88 genetic IVs fulfilling the genome-wide significance and LD of r^2^ > 0.8 were selected. One incompatible SNP (rs140143) and four palindromic SNPs (rs10870077, rs12132349, rs1927681, and rs6466198) were removed. The remaining 83 SNPs were utilized for two-sample MR analysis. There were 123 independent IVs strongly associated with Crohn’s disease that were identified. Two incompatible and seven palindromic SNPs (rs11768997, rs140143, rs10878302, rs10956252, rs10995271, rs17388425, rs1927681, rs2847293, and rs35730213) were found after harmonizing the exposure and outcome GWAS. MR-PRESSO test result indicated that there were no outlier SNPs. Finally, 114 SNPs were used for the subsequent MR analysis. The general information of the chosen genetic IVs for ulcerative colitis and Crohn’s disease was listed in **Supplementary Tables S2, S3**.

### 3.2 The causal effect of IBD on HL

Malignant lymphomas are a heterogeneous group of cancers. Based on histopathological lesions, malignant lymphoma can be divided into HL and NHL. Even the different types of NHL exhibited significant heterogeneity in terms of pathological patterns, immunophenotypes, clinical manifestations, and treatment outcomes (16). Therefore, the outcome GWASs were defined as HL as well as four types of NHL including DLBCL, FL, Mature T/NK-cell lymphomas, and Other and unspecified types of NHL. Two-sample MR analysis results revealed that genetically determined liability to IBD had no causal effect on HL [OR_IVW_ = 1.048, 95% confidence interval (CI) 0.898–1.223, P = 0.551]. Similarly, the results of the subgroup analysis illustrated that neither ulcerative colitis (OR_IVW_ = 1.069, 95% CI 0.906–1.261, P = 0.430) nor Crohn’s disease (OR_IVW_ = 0.955, 95% CI 0.837–1.091, P = 0.501) increased the risk of HL (**Supplementary Table S4**).

### 3.3 The causal effect of IBD on NHL

**Figure 3:**
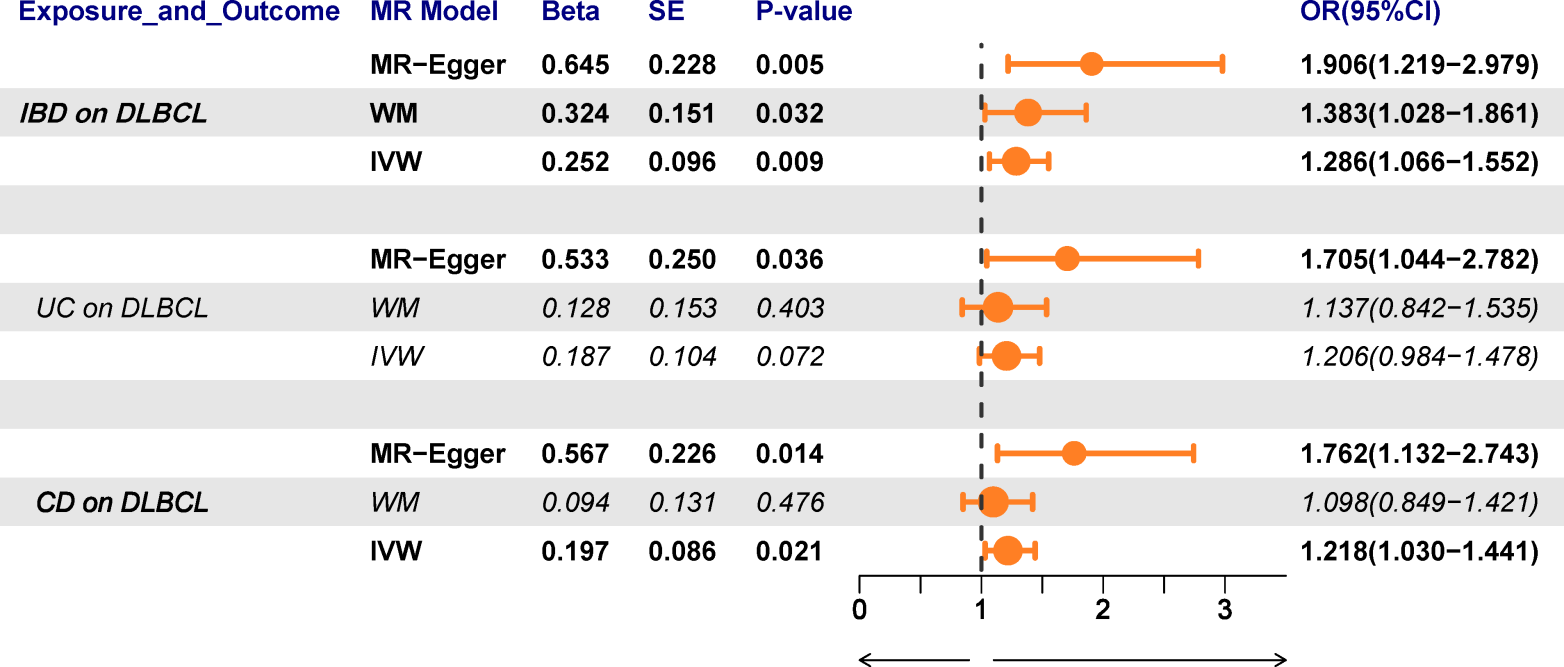
The forest plots revealed the causal association of inflammatory bowel disease (IBD) with diffuse large B-cell lymphoma (DLBCL). The results of the subgroup analysis showed that Crohn’s disease rather than ulcerative colitis had a causal effect on DLBCL.

**Figure 4:**
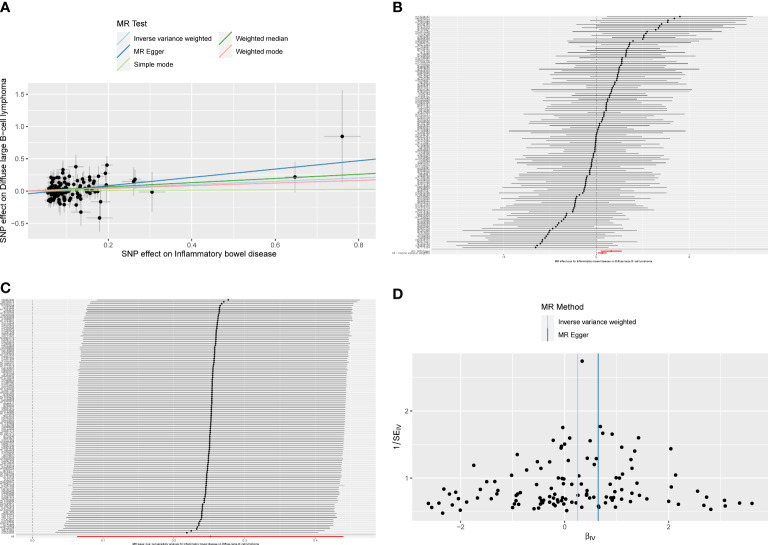
**(A)**The scatter plot from genetically predicted inflammatory bowel disease (IBD) on diffuse large B-cell lymphoma (DLBCL). **(B)** The forest plot of causality effect sizes of both single and merged single-nucleotide polymorphisms (SNPs) for IBD on DLBCL. **(C)** The results of leave-one-out methods for sensitivity analysis. **(D)** The funnel plot from genetically predicted IBD on DLBCL.

The overall causal estimates proved the causality of IBD on DLBCL, which was visualized in the forest plot **(** **Figure 3**). Patients with IBD have a 28.6% increased risk of developing DLBCL (OR_IVW_ = 1.286, 95% CI 1.066–1.552, P = 0.009). Moreover, the causal effect estimates of IBD on DLBCL from the other two MR models were consistent (OR_MR-Egger_ = 1.906, 95% CI 1.219–2.979, P = 0.005; OR_WM_ = 1.383, 95% CI 1.028–1.861, P = 0.032). The scatter plot (**Figure 4A**) also demonstrated the elevated risk of DLBCL in patients with IBD. The forest plot showed the effect sizes and their 95% CI for each of the independent SNPs in the IBD GWAS, as well as the overall causal estimates from the MR-Egger and IVW models (**Figure 4B**). The Cochran’s Q test results suggested that no significant heterogeneity was observed in the MR study (P_MR-Egger_ = 0.413, P_IVW_ = 0.350). After the MR-Egger intercept test, the P-value was greater than 0.05, indicating that there was no gene directional pleiotropy. The sensitivity analysis used the leave-one-out method, as shown in **Figure 4C**, and the results indicated that the conclusion of causality estimation of IBD on DLBCL remained stable and reliable when any of the selected SNPs was deleted. The funnel plot (**Figure 4D**) was approximately symmetric, suggesting the absence of directional pleiotropy.

**Figure 5:**
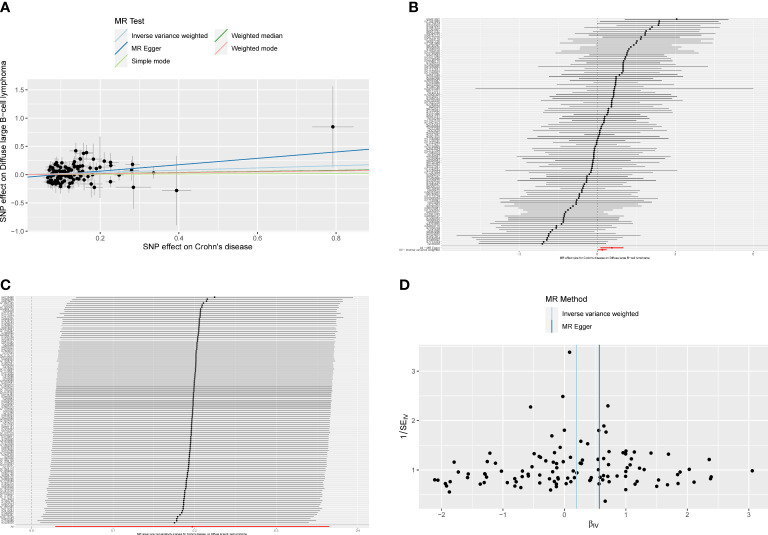
**(A)**The scatter plot from genetically predicted Crohn’s disease on diffuse large B-cell lymphoma (DLBCL). **(B)** The forest plot of causality effect sizes of both single and merged single-nucleotide polymorphisms (SNPs) for Crohn’s disease on DLBCL. **(C)** The results of leave-one-out methods for sensitivity analysis. **(D)** The funnel plot from genetically predicted Crohn’s disease on DLBCL.

The results of the subgroup analysis showed that ulcerative colitis had no causal effect on DLBCL (OR_IVW_ = 1.206, 95% CI 0.984–1.478, P = 0.072). However, for Crohn’s disease patients, there was an increased risk of developing DLBCL with an OR of 1.218 (IVW, 95% CI 1.030–1.441, P = 0.021; OR_MR-Egger_ = 1.762, 95% CI 1.132–2.743, P = 0.014; **Figure 5A**). The forest plot was showed in **Figure 5B**. Heterogeneity and pleiotropy were not detected in the causality estimates of ulcerative colitis on DLBCL. The result of the leave-one-out method confirmed that the overall causality results were reliable. The funnel plot was roughly symmetrical (**Figures 5C, D**).

As for the other types of NHL, IBD had no causal association with them. The P-values for all three MR methods were greater than 0.05 and can be available in **Supplementary Table S5**.

## 4 Discussion

IBDs are a group of chronic inflammatory diseases of the gastrointestinal tract that includes two major entities: ulcerative colitis and Crohn’s disease. The clinical manifestations of IBD patients consist of a range of symptoms associated with chronic inflammation of the intestinal tract, including fever, abdominal pain, diarrhea, anemia, and weight loss (17). Current therapeutic options for IBD include corticosteroids, 5-aminosalicylic acid (5-ASA)-based drugs (sulfasalazine and mesalazine), immunomodulators (azathioprine, 6-mercaptopurine, and methotrexate), biologicals (anti-TNF-α monoclonal antibodies: adalimumab and infliximab, vedolizumab and ustekinumab), fecal microbiota transplantation, and surgical treatment (18). However, while these treatment strategies alleviated symptoms and induced and maintained remission in patients with IBD, several observational cohort studies have reported that patients receiving thiopurines, anti-TNF monotherapy, or a combination of both drugs were at risk of developing lymphoma (19–21). In addition, a large Swedish cohort study revealed that developing IBD in childhood was associated with a high incidence of cancers compared to the matched general population comparators [hazard ratio (HR) = 2.2, 95% CI 2.0–2.5] and that the elevated risk does not decrease over time (22). Similarly, a large Israel nationwide cohort study concluded that pediatric-onset IBD patients had an increased risk of developing cancer and lymphoproliferative disorders, regardless of the therapies (23). In fact, it is difficult to distinguish whether it is the chronic inflammatory disease activity itself or the treatment regimen, including immunosuppressive drugs and biological agents, that increases a patient’s risk of malignant lymphoma. In any case, the risk of lymphoproliferative disorders such as malignant lymphoma has become a major concern for internists managing patients with IBD (24).

On the other hand, it is widely believed that infection and immune factors play an important role in the pathogenesis of lymphoma. Autoimmune diseases such as systemic lupus erythematosus (SLE), rheumatoid arthritis (RA), primary Sjögren’s syndrome (pSS), and ulcerative colitis have been observed to be associated with an increased risk of NHL and are potential risk factors for malignant lymphoma (25–27). Therefore, in general, studies on the causality of IBD and malignant lymphoma have been receiving extensive attention. Unfortunately, there is no consensus on the causal association between the two. There are a few limitations in observational studies and randomized controlled trials (RCTs), but it is good to see that GWASs have been gradually carried out in IBD and lymphoma in recent years, and some genetic variant susceptibility loci for the two diseases have been identified, allowing MR studies to be performed to elucidate the causal association (15, 28). This is the first two-sample MR study on the causal effect of IBD itself on malignant lymphoma.

In this study, MR analysis was performed to assess the evidence of causal association of IBD and malignant lymphoma. There were 124, 83, and 114 strongly correlated and independent SNPs identified for IBD, ulcerative colitis, and Crohn’s disease, respectively. MR results showed a 28.6% elevated risk of DLBCL in IBD patients, suggesting that IBD is a risk factor for developing DLBCL independent of treatment strategies (OR_IVW_ = 1.286, 95% CI 1.066–1.552, P = 0.009). The results of subgroup analysis demonstrated the causal effect of Crohn’s disease on DLBCL (OR_IVW_ = 1.218, 95% CI 1.030–1.441, P = 0.021) rather than ulcerative colitis (OR_IVW_ = 1.206, 95% CI 0.984–1.478, P = 0.072). Moreover, the present study did not find a causal association of IBD with HL and other types of NHL.

Previous research has been devoted to exploring the potential mechanisms underlying the causal relation between IBD and DLBCL, which were summarized as follows. IBD is a group of immune-mediated chronic nonspecific inflammatory disorders of the gastrointestinal tract. Chronic inflammatory response and immune dysregulation play an important role in the development and progression of IBD (29, 30). A plethora of studies exploring the positive association of autoimmune and inflammatory diseases with malignant lymphoma development confirmed that B cells are exposed to various types of antigens (autologous, viral, and microbial antigens) in the context of autoimmunity and inflammation, activating B-cell receptor signaling pathways and sustaining B-cell proliferation, response, and clonal amplification. In turn, a few inherent genetic instability events in lymphocytes including IGHV-IGHD-IGHJ recombination, somatic hypermutation, and class-switch recombination are at increased risk during B-cell maturation. This ultimately leads to the development of malignant lymphoma (31, 32). More specific relevant molecular mechanisms were elucidated by Riva et al. (33). The study found that IL1R8, a member of the interleukin-1 receptor family, exerts anti-inflammatory responses upon activation. Downregulation of IL1R8 is associated with heightened inflammatory response and autoimmunity. IL1R8 was downregulated in human DLBCL cells and was associated with worse overall survival. Experiments have demonstrated that IL1R8 deficiency is associated with increased lymphatic proliferation and transformation, thus involved in the pathogenesis of autoimmune-associated B-cell lymphoma.

The present study demonstrates a causal relationship between IBD, especially Crohn’s disease, and DLBCL. However, it has a few limitations. On the one hand, the population enrolled in this study was mainly European, so the causal relation between IBD and DLBCL is still unknown in other populations. On the other hand, observational studies have also reported that some specific treatment regimens for IBD also increased the risk of malignant lymphoma. Limited by the lack of GWAS related to IBD treatment modalities, the causal relationship could not be verified by MR studies, and subsequent relevant GWAS and MR studies are needed.

## 5 Conclusions

From this MR study, it can be concluded that IBD is a potential risk factor for DLBCL. The results of the subgroup analysis suggest that Crohn’s disease, rather than ulcerative colitis, increases the risk of developing malignant lymphoma. The mechanism underlying this causal link remains to be further investigated. It reminds internists to pay more attention to the causal association between IBD and malignant lymphoma during clinical practice.

## Data availability statement

The original contributions presented in the study are included in the article/**Supplementary Material**. Further inquiries can be directed to the corresponding authors.

## Ethics statement

This study used publicly available data from studies on human experimentation that have approved by their respective institutional review boards. No additional ethical approval was required for this MR study.

## Author contributions

LY designed the study. CL and QC wrote the article. LX collected and processed the data. JL was responsible for the Figures. CW and LY were responsible for revising the article. HT provided helpful guidance and made great contributions in the process of manuscript revision. All authors contributed to the article and approved the submitted version.
